# Deep infiltrating ureteral endometriosis with catamenial hydroureteronephrosis: a case report

**DOI:** 10.1186/s13256-017-1518-6

**Published:** 2017-12-13

**Authors:** Hyun Jung Lee, Yoon Soon Lee

**Affiliations:** 1 Department of Obstetrics and Gynecology, School of Medicine, Kyungpook National University, 130, Dongdeok-ro, Jung-gu, Daegu, 41944 Republic of Korea; 2 Department of Obstetrics and Gynecology, School of Medicine, Kyungpook National University, 807, Hoguk-ro, Buk-gu, Daegu, 41404 Republic of Korea

**Keywords:** Ureteral endometriosis, Deep infiltrating endometriosis, Catamenial hydroureteronephrosis

## Abstract

**Background:**

This aim of this case report is to raise awareness of ureteral endometriosis in women of reproductive age with hydronephrosis in the absence of urolithiasis to enable early diagnosis and prevent loss of renal function.

**Case presentation:**

A 44-year-old Asian woman presented with a 4-year history of cyclic right flank pain and right hydronephrosis during menstruation. Despite several evaluations by physicians, including gynecologists, the cause of her symptoms was not diagnosed. On transvaginal ultrasonography, the uterus was observed deviated to the right, with a nodular lesion at the right uterosacral ligament, and the right ovary was attached to the uterus with no apparent cystic lesion. Magnetic resonance imaging showed a mass in the right uterine wall and mild wall thickening with delayed enhancement of the right distal ureter. Right ureteral endometriosis was suspected. Diagnostic laparoscopy revealed narrowing of the distal right ureter between the right uterosacral ligament and the right ovary with adhesions caused by deep infiltrating endometriosis. The adhesion bands and infiltrating endometriosis around the right ureter were dissected.

**Conclusions:**

The nonspecific symptoms of ureteral endometriosis can result in incorrect diagnosis, with renal damage as a result of prolonged hydronephrosis. A high index of suspicion and use of imaging modalities enable earlier diagnosis and preservation of renal function.

## Background

Endometriosis is defined as the presence of endometrial glandular and stromal tissue outside the uterus. It is a chronic, estrogen-dependent gynecological disease that affects 6–10% of reproductive age women [1]. Endometriosis is generally classified according to morphology and localization as ovarian, superficial peritoneal, or deep infiltrating endometriosis (DIE). DIE may involve the rectosigmoid colon, the rectovaginal space, uterosacral ligaments, and the bowel or urinary tract [2]. Among these, urinary tract involvement by endometriosis has been observed in 1% of women with pelvic endometriosis, and the bladder is most commonly affected [3]. Ureteral endometriosis is relatively uncommon and is estimated to occur in about 0.08–1% of patients with endometriosis [4, 5]. However, the diagnosis of ureteral endometriosis may be delayed, leading to serious consequences such as urinary tract obstruction and silent loss of renal function. The risk of silent renal loss due to DIE is reportedly as high as 25–50% [6].

We report the case of a 44-year-old Asian woman with right ureteral endometriosis who presented to our institution with catamenial right flank pain but no other symptoms of endometriosis. This case was considered important because of the unusual location and the absence of endometriosis symptoms other than catamenial right flank pain and hydroureteronephrosis. The aim of this case report is to raise awareness of ureteral endometriosis in women of reproductive age with hydronephrosis in the absence of urolithiasis to enable early diagnosis and prevent loss of renal function.

## Case presentation

**Fig. 1:**
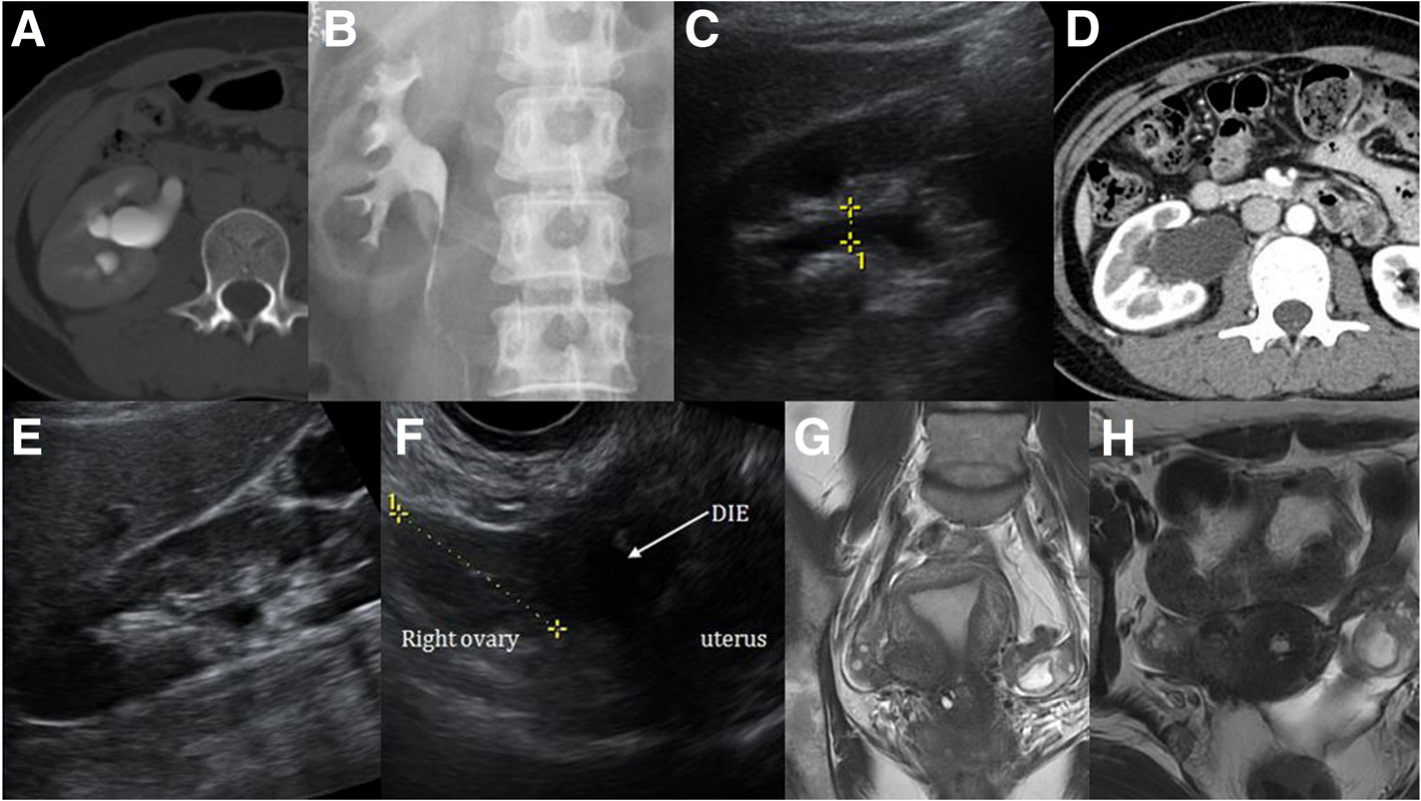
Computed tomography showed right hydronephrosis due to distal ureteral narrowing 4 years prior (**a**). After balloon dilation, the patient’s right hydronephrosis was relieved (**b**). Transabdominal ultrasonography (**c**) and computed tomography (**d**) demonstrated right hydronephrosis due to narrowing of the distal ureter at the start of menstruation. After menstruation, hydronephrosis was relieved (**e**). Transvaginal ultrasonography revealed uterine deviation to the right and a nodular lesion at the right uterosacral ligament (**f**). Magnetic resonance imaging revealed suspected deep infiltrating endometriosis near the right uterus and ovary, with adhesions around the distal ureter (**g** and **h**)

A 44-year-old Asian woman presented to our institution with right flank pain. She reported recurrent right flank pain during menstruation of 4 years’ duration. Shortly after onset, she visited an emergency center and was diagnosed with right hydroureteronephrosis on the basis of computed tomography (CT) at another hospital (Fig. 1a). She underwent balloon dilation of the right distal ureter. Her right hydronephrosis was relieved, and the final diagnosis was idiopathic right distal ureteral stricture (Fig. 1b). She subsequently had mild cyclic right flank pain and visited numerous physicians, including gynecologists, but the cause of her symptoms was not diagnosed. Two weeks before visiting our hospital, her right flank pain was acutely aggravated during menstruation. She visited another hospital and was diagnosed on the basis of ultrasonography (US) and CT with right hydronephrosis due to right distal ureter stricture; however, her hydronephrosis resolved after 1 week (Fig. 1c and d).

The patient denied any menstrual cycle irregularities or history of endometriosis; chronic disease; allergies; hospitalizations; or past surgical history, including abdominal and gynecological surgeries. The result of a comprehensive review of systems was negative. Routine laboratory test results, including urinalysis, were all within normal limits. Transabdominal US revealed improved right hydronephrosis (Fig. 1e) compared with the previous CT findings. On transvaginal US, the uterus was deviated to the right with a nodular lesion at the right uterosacral ligament; the right ovary was attached to the uterus, with no apparent cystic lesion (Fig. 1f). Magnetic resonance imaging (MRI) showed a 2.8-cm heterogeneous mass in the right uterine wall with a tiny implant in the serosa of the posterior uterine wall. Mild wall thickening with delayed enhancement and mild dilation of the right distal ureter were observed, suggesting fibrotic change around the right distal ureter (Fig. 1g, h). These radiologic findings were consistent with ureteral DIE.

**Fig. 2:**
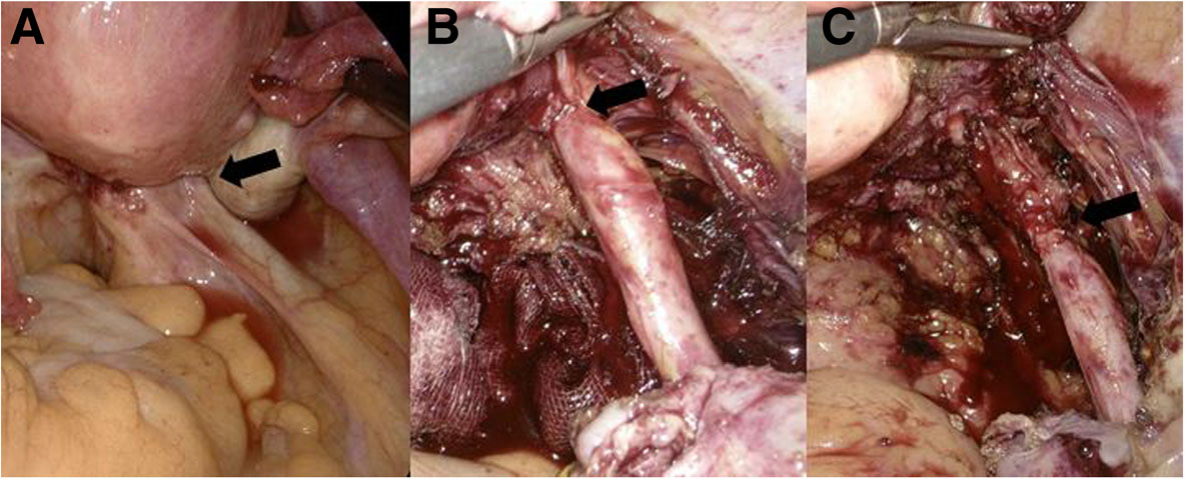
The distal right ureter (*arrow*) was narrowed because of deep infiltrating endometriosis (**a**). The ureter was dissected in the direction of the area where the lateral extension of deep infiltrating endometriosis surrounded the ureter (**b**). After dissection of the uterine artery, the ureter was free to its lowest segment (**c**)

Diagnostic laparoscopy revealed narrowing of the distal right ureter between the right uterosacral ligament and right ovary, with adhesions caused by DIE (Fig. 2a). Dense adhesive fibrotic bands around the right distal ureter were dissected after resection of the right uterosacral ligament (Fig. 2b). Resection of the ipsilateral uterine artery crossing the distal part of the ureter was performed with ureterolysis to its most distal segment (Fig. 2c). A double-J stent was inserted into the right ureter to prevent possible obstruction. The pathologic report confirmed DIE of the right uterosacral ligament. The patient also received gonadotropin-releasing hormone agonists and is now free of symptoms.

## Discussion

Ureteral endometriosis has intrinsic and extrinsic pathological types [7]. The intrinsic type is characterized by invasion of endometriotic glands and stroma in the ureteral wall, and it is less common than extrinsic ureteral endometriosis, which is caused by external compression by surrounding DIE. According to Stanley *et al*. [8], endometriosis of the ureter usually arises by extension from pelvic endometriosis. The distal segment of the left ureter is more frequently involved because of the adjacent reproductive organs [9].

Symptoms of endometriosis, depending on the location and extent of lesions and severity of disease, can lead to pain, cyclic hematuria, and infertility caused by extensive adhesions and distortion of anatomy. Among these, cyclic hematuria is pathognomonic for urinary tract involvement by endometriosis and presents in 20% of cases with bladder endometriosis [10]. Symptoms are especially aggravated during menstruation because blood is increased within the involved organs and can distend the surrounding tissue or peritoneum [11]. If a reproductive age woman presents with these symptoms and no documented infection, endometriosis should be suspected.

The symptoms of urinary tract endometriosis are nonspecific and can be confusing. Ureteral endometriosis is asymptomatic in as many as 50% of patients [12]. Because of nonspecific symptoms, insufficient preoperative evaluation, misinterpretation of imaging findings, or nonspecific imaging findings, ureteral endometriosis is suspected before surgery in only 40% of patients [13]. Incorrect diagnosis of ureteral endometriosis can lead to obstructive uropathy and permanent renal failure.

Our patient’s case of right distal ureteral endometriosis caused extrinsic compression with fibrous adhesion formation by DIE of the right uterosacral ligament. The only symptom was right flank pain aggravated during the menstrual period with no symptoms suggesting endometriosis, such as dysmenorrhea, dyspareunia, or cyclic urinary pain. Pelvic and vaginal examination findings were also nonspecific. The only clue to endometriosis was the periodic right flank pain and hydronephrosis on radiological examination. Given the history and concern about the possibility of endometriosis, transvaginal US was performed and revealed a suspicious nodular lesion at the right uterosacral ligament, which correlated with a heterogeneous mass on MRI. Right ureteral wall thickness with delayed enhancement was confirmed during surgery as fibrotic change associated with endometriosis.

Surgery is necessary in patients with ureteral endometriosis who have persistent symptoms and/or hydroureteronephrosis. The main goals of surgery are preservation of renal function, relief of obstruction, and prevention of recurrence. The surgical modalities include ureterolysis, ureterostomy, distal ureterectomy, and ureteral reimplantation, according to the extent, severity, and type of disease. Ureterolysis using a laparoscopic or open approach is indicated in patients with extrinsic ureteral endometriosis if there is an extrinsic lesion < 3 cm and/or nonobstructive ureteral involvement [14]. Laparoscopic ureterolysis, DIE resection, and double-J ureteral stent insertion were performed in our patient because she had extrinsic distal ureteral endometriosis without complete obstruction. An individualized therapy plan, including hormone or surgical therapy, should be considered according to patient age, type of ureteral endometriosis, extent of disease, degree of hydronephrosis, and renal function.

## Conclusions

Nonspecific symptoms and incorrect diagnosis of ureteral endometriosis can result in renal damage as a result of prolonged hydronephrosis. Therefore, physicians should suspect ureteral endometriosis in reproductive age women with unilateral or bilateral distal ureteral obstruction of uncertain cause. A high index of suspicion and use of imaging modalities enable earlier diagnosis, preservation of renal function, and improved prognosis.

## References

[1] Bulun SE. Endometriosis. N Engl J Med. 2009;360:268–79. 10.1056/NEJMra0804690 19144942

[2] Traşcă ET, Traşcă E, Tiţu A, Riza ML, Busuioc I. Ureteral stenosis due to endometriosis. Rom J Morphol Embryol. 2012;53:433–7. 22732821

[3] Giudice LC, Kao LC. Endometriosis. Lancet. 2004;364:1789–99. 10.1016/S0140-6736(04)17403-5 15541453

[4] Nezhat C, Nezhat F, Nezhat CH, Nasserbakht F, Rosati M, Seidman DS. Urinary tract endometriosis treated by laparoscopy. Fertil Steril. 1996;66:920–4. 8941055

[5] Stillwell TJ, Kramer SA, Lee RA. Endometriosis of ureter. Urology. 1986;28:81–5. 10.1016/0090-4295(86)90092-0 3739125

[6] Watanabe Y, Ozawa H, Uematsu K, Kawasaki K, Nishi H, Kobashi Y. Hydronephrosis due to ureteral endometriosis treated by transperitoneal laparoscopic ureterolysis. Int J Urol. 2004;11:560–2. 10.1111/j.1442-2042.2004.00828.x 15242370

[7] Gehr TW, Sica DA. Case report and review of the literature: ureteral endometriosis. Am J Med Sci. 1987;294:346–52. 10.1097/00000441-198711000-00009 3321982

[8] Stanley Jr KE, Utz DC, Dockerty MB. Clinically significant endometriosis of the urinary tract. Surg Gynecol Obstet. 1965;120:491–8. 14262046

[9] Yohannes P. Ureteral endometriosis. J Urol. 2003;170:20–5. 10.1097/01.ju.0000054836.32660.9e 12796637

[10] Batler RA, Kim SC, Nadler RB. Bladder endometriosis: pertinent clinical images. Urology. 2001;57:798–9. 10.1016/s0090-4295(00)01114-6 11306413

[11] Kumar S, Tiwari P, Sharma P, Goel A, Singh JP, Vijay MK, *et al.* Urinary tract endometriosis: review of 19 cases. Urol Ann. 2012;4:6–12. 10.4103/0974-7796.91613 PMC3271455 22346093

[12] Gabriel B, Nassif J, Trompoukis P, Barata S, Wattiez A. Prevalence and management of urinary tract endometriosis: a clinical case series. Urology. 2011;78:1269–74. 10.1016/j.urology.2011.07.1403 21962747

[13] Ponticelli C, Graziani G, Montanari E. Ureteral endometriosis: a rare and underdiagnosed cause of kidney dysfunction. Nephron Clin Pract. 2010;114:c89–93. 10.1159/000254380 19887828

[14] Maccagnano C, Pellucchi F, Rocchini L, Ghezzi M, Scattoni V, Montorsi F, *et al.* Ureteral endometriosis: proposal for a diagnostic and therapeutic algorithm with a review of the literature. Urol Int. 2013;91:1–9. 10.1159/000345140 23689345

